# Anemoside B4 protects against *Klebsiella pneumoniae*- and influenza virus FM1-induced pneumonia via the TLR4/Myd88 signaling pathway in mice

**DOI:** 10.1186/s13020-020-00350-w

**Published:** 2020-07-02

**Authors:** Jia He, Renyikun Yuan, Xiaolan Cui, Yushun Cui, Shan Han, Qin-Qin Wang, Yangling Chen, Liting Huang, Shilin Yang, Qiongming Xu, Yonghui Zhao, Hongwei Gao

**Affiliations:** 1 College of Pharmacy, Guangxi University of Chinese Medicine, Nanning, 530000 China; 2 Guangxi Engineering Technology Research Center of Advantage Chinese Patent Drug and Ethnic Drug Development, Nanning, 530020 China; 3 Jiangxi University of Traditional Chinese Medicine, Nanchang, 330004 China; 4 Institute of Chinese Materia Medica, China Academy of Chinese Medical Sciences, Beijing, 100700 China; 5 College of Pharmaceutical Science, Soochow University, Suzhou, 215123 China; 6 Qingdao Central Hospital, The Second Affiliated Hospital of Qingdao University, Qingdao, 266109 China

**Keywords:** Anemoside B4, Pneumonia, *Klebsiella pneumoniae*, Influenza virus FM1, TLR4/Myd88

## Abstract

**Background:**

Pneumonia refers to the inflammation of the terminal airway, alveoli and pulmonary interstitium, which can be caused by pathogenic microorganisms, physical and chemical factors, immune damage, and drugs. Anemoside B4, the major ingredient of *Pulsatilla chinensis* (Bunge) Regel, exhibited anti-inflammatory activity. However, the therapeutic effect of anemoside B4 on pneumonia has not been unraveled. This study aims to investigate that anemoside B4 attenuates the inflammatory responses in *Klebsiella pneumonia* (KP)- and influenza virus FM1 (FM1)-induced pneumonia mice model.

**Methods:**

The network pharmacology and molecular docking assays were employed to predict the targets of anemoside B4’s treatment of pneumonia. Two models (bacterial KP-infected mice and virus FM1-infected mice) were employed in our study. BALB/c mice were divided into six groups: control, model group (KP-induced pneumonia or FM1-induced pneumonia), anemoside B4 (B4)-treated group (2.5, 5, 10 mg/kg), and positive drug group (ribavirin or ceftriaxone sodium injection). Blood samples were collected for hematology analysis. The effects of B4 on inflammation-associated mediators were investigated by Enzyme-linked immunosorbent assay (ELISA) and hematoxylin and eosin staining (HE) staining. Proteins expression was quantified by western blotting.

**Results:**

The network results indicated that many pro-inflammatory cytokines such as tumor necrosis factor α (TNF-α), interleukin-1β (IL-1β), and interleukin-6 (IL-6) participated in anemoside B4’s anti-inflammatory activity. The counts of neutrophil (NEU) and white blood cell (WBC), the level of myeloperoxidase (MPO), and the release of pro-inflammatory cytokines TNF-α, IL-1β, and IL-6 increased by KP or FM1 infection, which were reversed by anemoside B4. In addition, anemoside B4 significantly suppressed the FM1-induced expression of toll-like receptor 4 (TLR4), myeloid differential protein-88 (MyD88), and myeloid differentiation protein-2 (MD-2), which were further validated by molecular docking data that anemoside B4 bound to bioactive sites of TLR4. Therefore, anemoside B4 exhibited a significant therapeutic effect on pneumonia via the TLR4/MyD88 pathway.

**Conclusion:**

Our findings demonstrated that anemoside B4 attenuates pneumonia via the TLR4/Myd88 signaling pathway, suggesting that anemoside B4 is a promising therapeutic candidate for bacterial-infected or viral-infected pneumonia.

## Background

Pneumonia refers to the inflammation of the terminal airway, alveoli and pulmonary interstitium caused by many factors, of which typical symptoms include dry cough, chest pain, fever, and dyspnea [1, 2]. Pneumonia has been one of the main factors affecting people’s health and even life for a long time. With the introduction of antibiotics and vaccines in the twentieth century, the survival rate of related patients has been greatly improved [3]. However, pneumonia remains the leading cause of death in developing countries, as well as in elderly, young children and chronically ill patients [4]. *Klebsiella pneumoniae* is one of the most important opportunistic pathogens and iatrogenic pathogens, which is highly pathogenic to human beings [5]. Due to the abuse of various antimicrobial agents, the multi drug resistance of *Klebsiella pneumoniae* is widespread, which leads to great trouble for clinical treatment [6, 7]. Influenza virus is the representative of orthomyxoviridae, including human influenza virus and animal influenza virus [8, 9]. Influenza virus infection, whether seasonal or pandemic, often leads to pneumonia, which can cause serious health problems [10, 11]. For the treatment of viral pneumonia, apart from neuraminidase inhibitors and ribavirin, other antiviral drugs showed no significant effect on pneumonia [12]. Specifically, now corona virus COVID-19-induced pneumonia outbreaks world widely, leading to many deaths. As it stands now, there is not effective drugs for the treatment with pneumonia. Therefore, it is urgent to find new and effective drugs for treatment with pneumonia.

Network pharmacology is an emerging discipline that builds and analyzes biological networks based on systems biology to reveal the role of drugs and their mechanisms [13, 14]. By constructing a “component-target-pathway” research network, we can observe how drugs act on multiple targets at the same time, thereby regulating multiple signal pathways, comprehensively reveal their drug efficacy network, and explain their mechanism of action [15]. This provides a certain guiding significance for our work.

During pneumonia process, white blood cell (WBC) and neutrophil (NEU) counts are always increased [16]. In addition, myeloperoxidase (MPO), an enzyme secreted by leukocytes, is present in myeloid cells, which catalyzes the formation of a variety of active oxidants during the occurrence and development of pneumonia [17]. The role of TLRs in the signaling pathway of inflammation and related diseases is highly valued [18]. When induced by external stimuli such as viruses or bacteria, the adaptor protein myeloid differentiation protein-2 (MD2) directly binds and recognizes stimuli forming discrete complex, and associates non-covalently toll-like receptor 4 (TLR4) to form the final activated heterodimer that in its turn starts the intracellular signal [19]. After that, myeloid differentiation primary response 88 (Myd88) is recruited to TLR4, which then activates downstream pathways and results in the pro-inflammatory cytokine release, such as tumor necrosis factor-α (TNF-α), interleukin-6 (IL-6), and interleukin-1β (IL-1β) [20]. This suggests that TLR4/MyD88 signaling pathway may be an effective therapeutic target for many inflammatory diseases.

Traditional Chinese medicine has great potential in the treatment of many inflammatory and immunoregulatory diseases [21, 22]. *Pulsatilla chinensis* (Bunge) Regel, a traditional Chinese herb, has the functions of clearing away heat and detoxification, stopping dysentery and drying dampness, and has a good therapeutic effect on bacteria, virus infection and malignant tumors in clinic [23]. Recent studies have shown that triterpenoid saponins are the main factors affecting the pharmacological activities in this herb. Anemoside B4, one of the main monomer components of *Pulsatilla chinensis* (Bunge) Regel, can inhibit the pathogenesis of acute kidney injury caused by cisplatin and improve renal function, which protective effects may be associated with its anti-inflammatory activities [24]. However, there are not papers involved in the therapeutic effect of B4 on pneumonia. In the present study, we used *Klebsiella pneumoniae*- or influenza virus FM1-induced pneumonia model to investigate the anti-inflammatory effects and mechanisms of anemoside B4 in vivo.

## Methods

### Reagents and chemicals

*Klebsiella pneumoniae* (BNCC-102997) was purchased from Beijing Beina Chuanglian Biotechnology Research Institute (Beijing, China). Influenza virus FM1 strain was provided by ABSL-2 laboratory, Institute of traditional Chinese medicine, Chinese Academy of Sciences (Beijing, China). Antibodies against MyD88 (#4283), TLR4 (#14358) were obtained from Cell Signaling (Beverly, MA, USA), and antibody against MD2 (#24182) was obtained from Abcam (Cambridge, MA, England). Ribavirin (20160308) was purchased from Solarbio (Beijing, China). Ceftriaxone sodium Injection was purchased from ReYoung Pharmaceutical Co., Ltd (Shandong, China). IL-1β, IL-6, and TNF-α ELISA kits were obtained from Neobioscience (Shenzhen, China). MPO kit (A044-1-1) was purchased from Nanjing Jiancheng Bioengineering Institute (Nanjing, China). Physiological saline for injection (L219012211) was purchased from Sichuan Kelun Pharmaceutical Co., Ltd. (Chengdu, China).

### Animals

The study was approved by the Ethics Committee on Laboratory Animal Management of Guangxi University of Chinese Medicine (Approval Document No. SYXK-GUI-2019-0001). Virus experiment was carried out in ABSL-2 laboratory, Institute of traditional Chinese medicine, Chinese Academy of Sciences. All animals received humane care according to the Local Guide for the Care and Use of Laboratory Animals of Guangxi University of Chinese Medicine. Healthy BALB/c mice (male and female, 6–8 weeks-old and weighing 18–22 g) were purchased from Beijing Vital River Laboratory Animal Technology Co., Ltd. (Beijing, China) and acclimated for 3 days (animal license #: SCXK 2016–0006). All animals were housed under standard specific pathogen-free (SPF) conditions and given free access to food and water with a controlled temperature (25 °C) and humidity (50%).

### Preparation of anemoside B4

The dried roots of *Pulsatilla chinensis* (Bunge) Regel (10 kg) were powered into 100 mesh, which were further extracted by 70% ethanol to obtain liquid extract after removing the ethanol. By adding proper hot water, the extract was sequentially extracted with petroleum ether, dichloromethane, ethyl acetate, and *n*-butyl alcohol to get 24.2 g, 102.5 g, 34.2 g, and 685.2 g extract, respectively. The *n*-butyl alcohol (685.2 g) extract was further separated on a column packed with macroporous adsorption resins by gradient eluting with ethanol and water (100:0, 30:70, 60:40, 90:10) to yield four fractions: Fr. 1–Fr. 4. Fr. 3 (180.2 g) was further separated by using medium pressure liquid chromatography (MPLC) with ODS column first. Then Sephadex LH-20 gel column eluted with MeOH and semi-preparative HPLC with C18 column at a flow rate of 2 mL/min eluted with MeOH (60%) were employed for further purification to yield anemoside B4 (42.2 g). The purity of anemoside B4 was investigated by HPLC assay.

### *Klebsiella pneumoniae*-induced bacterial pneumonia of mice

All the male mice were divided randomly into six groups (n = 20 per group): control group, model group, anemoside B4-treated group (2.5, 5, 10 mg/kg) and ceftriaxone sodium-treated group (200 mg/kg). In addition to the control group, the other five groups were administrated with *Klebsiella pneumoniae* (1.04 × 10^9^ CFU/mL, 50 μL/per mouse) by non-invasive intratracheal (i.t.) instillation into lung of mice. Mice were treated with anemoside B4 (i.v.) at the point of time 0, 3, 24, 48, 72 h or ceftriaxone sodium (i.v.) at the point of time 0 h. 72 h after *Klebsiella pneumoniae* treatment, all experimental mice were sacrificed by dislocation (Fig. 3a). Blood samples were collected for measurement of levels of WBC, NEU, and pro-inflammatory cytokines. The parts of lung samples were collected and kept at − 80 °C for western blot assay. Bronchoalveolar lavage fluid (BALF) was also taken for pro-inflammatory cytokines.

### Influenza virus FM1-induced viral pneumonia of mice

All the mice were divided randomly into six groups (n = 20 per group, half male and female): control group, model group, anemoside B4-treated group (2.5, 5, 10 mg/kg) and ribavirin-treated group (40 mg/kg). In addition to the control group, the other five groups were administrated with influenza virus FM1 (The virus stock solution was diluted 500 times with normal saline solution) by non-invasive intratracheal (i.t.) instillation into lung of mice. Each group of mice was administrated (i.v.) with anemoside B4 (2.5, 5, 10 mg/kg) or ribavirin (40 mg/kg) at the point of time 0, 24, 48, 72, and 96 h. 120 h after treatment of influenza virus FM1, all experiment mice were sacrificed by dislocation (Fig. 5a). Blood samples and BALF were collected for pro-inflammatory molecules. The other of lung samples were collected and kept at − 80 °C for western blot assay.

### Network pharmacology predicts the mechanism of B4 in treating pneumonia

Systematic pharmacology software was used to predict the underlying targets and signal pathways of anemoside B4 in treating pneumonia. Through searching TTD and TCMSP database, the targets of anemoside B4 were determined, implying the underlying mechanisms of B4 acting on pneumonia.

### Hematology analysis

Blood was collected from mouse orbit. The blood sample was further processed with EDTA. White blood cell and neutrophil counts from blood were determined using an auto hematology analyzer (Mindray, Shenzhen, China).

### Enzyme-linked immunosorbent assay (ELISA)

The blood sample was placed at room temperature for 2 h and then centrifuged at 3000 rpm for 20 min to collect supernatant. The bronchoalveolar lavage fluid (BALF) was centrifuged at 1600 rpm for 10 min at 4 °C, and the supernatant was collected. The lung tissue was homogenized by a tissue grinder (TP-24, Jieling instrument manufacturing Tianjin Co., Ltd, Tianjin, China). The samples were centrifuged for 20 min at 3000 rpm. The supernatant was immediately stored at − 80 °C. The pro-inflammatory molecules IL-1β, TNF-α, and IL-6 of all the supernatants were investigated by ELISA kits following the manufacturer’s instructions.

### Pulmonary histopathology analysis

The lung tissue samples were fixed with 4% paraformaldehyde, dehydrated by alcohol gradient, and then transparently treated with xylene, paraffin-embedded and sectioned. The sections were stained with hematoxylin and eosin (H&E), and then the pathological changes of the tissue were observed by optical microscope (UOP, DSZ5000X, China).

### Western blotting analysis

Lung tissues were homogenized in RIPA buffer with 1% PMSF and 1% cocktail (Sigma-Aldrich, St. Louis, MO). The lysate was centrifuged at 15,000 rpm for 25 min at 4 °C and the supernatant was harvested. The protein concentrations were examined using a BCA protein kit (Thermofisher, Waltham, MA, USA). The denatured proteins were then separated by 10% SDS-PAGE gels and transferred to PVDF membrane (Millipore, Billerica, MA, USA). After blocking the PVDF membrane with 5% nonfat milk for 2 h, the PVDF membrane was incubated with primary antibodies (1:1000) at 4 °C overnight. After washed three times with TBST and incubated with secondary antibody (1:5000) for 2 h at room temperature, the protein band signals were detected with SuperSignal West Femto maximum sensitivity substrate (Pierce Biotechnology) in a ChemiDoc MP Imaging System (Bio-Rad, Hercules, CA, USA). GAPDH was used as a housekeeping protein.

### Molecular docking stimulation

The molecular docking data of B4 with TLR4-MD-2 (PDB code: 3FXI) was performed in Ledock (http://www.lephar.com). TLR4-MD-2 structures were obtained from RCSB Protein Data Bank (PDB code: 3FXI) [25].

### Statistical analysis

Data are presented as mean ± SD. All experiments were repeated at least three independent times. Data were normally distributed and analyzed by one-way-ANOVA by Graph Pad Prism 7 software (Microsoft, Seattle, WA, USA). A *p* value < 0.05 was considered to be statistically significant.

## Results

### The identification of anemoside B4

**Fig. 1:**
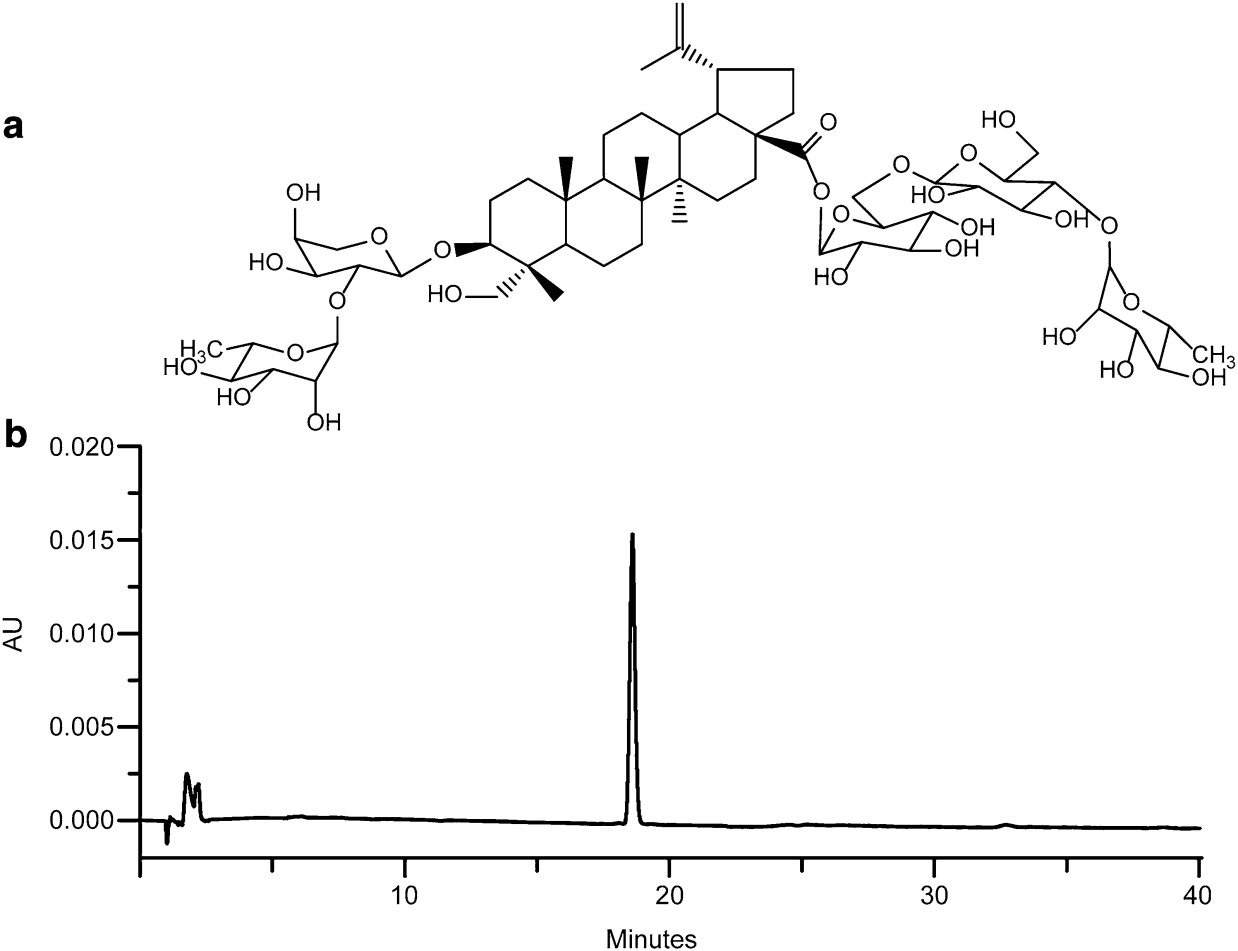
Chemical structure and chromatogram of enriched anemoside B4. **a** The chemical structure of anemoside B4. **b** Chromatogram of anemoside B4

As shown in Fig. 1a, the chemical structure of anemoside B4 was identified as 3-*O*-α-l-Rhamnopyranosyl-(1 → 2)-α-l-arabinofuranosyl-3β,23-dihydroxy lupinane-Δ20 (29)ene-28-*O*-α-l-Rhamnose-(1 → 4)-β-d-glucopyranose-(1 → 6)-β-d-glucopyranoside. The purity of anemoside B4 was over 98%, which was determined by HPLC assay (Fig. 1b).

### The target network of anemoside B4

**Fig. 2:**
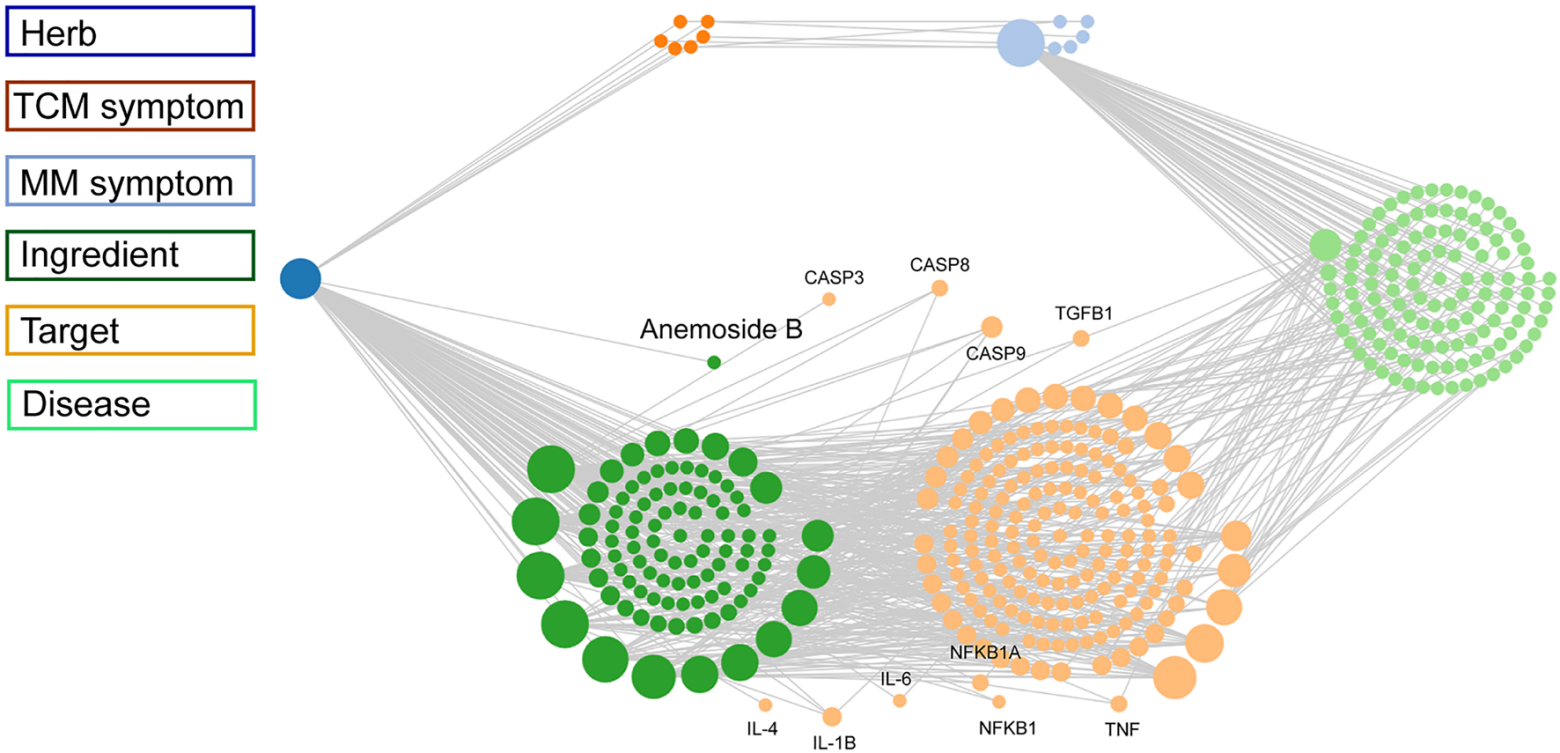
The network pharmacology of targets of anemoside B4 against pneumonia. The nodes in the network are colored and placed in different locations according to the source of the component, and the node size is customized according to its degree in the network

A network pharmacology-based strategy was proposed to elucidate the underlying multi-target mode of action of anemoside B4 against pneumonia (Fig. 2). This network consisted of 10 targets involved in inflammatory process [26, 27]. Specifically, three targets including IL-1β, IL-6, and TNF-α have been reported to be related to pneumonia, which were further validated in vivo.

### Anemoside B4 exhibits anti-inflammatory effects on KP-induced pneumonia

**Fig. 3:**
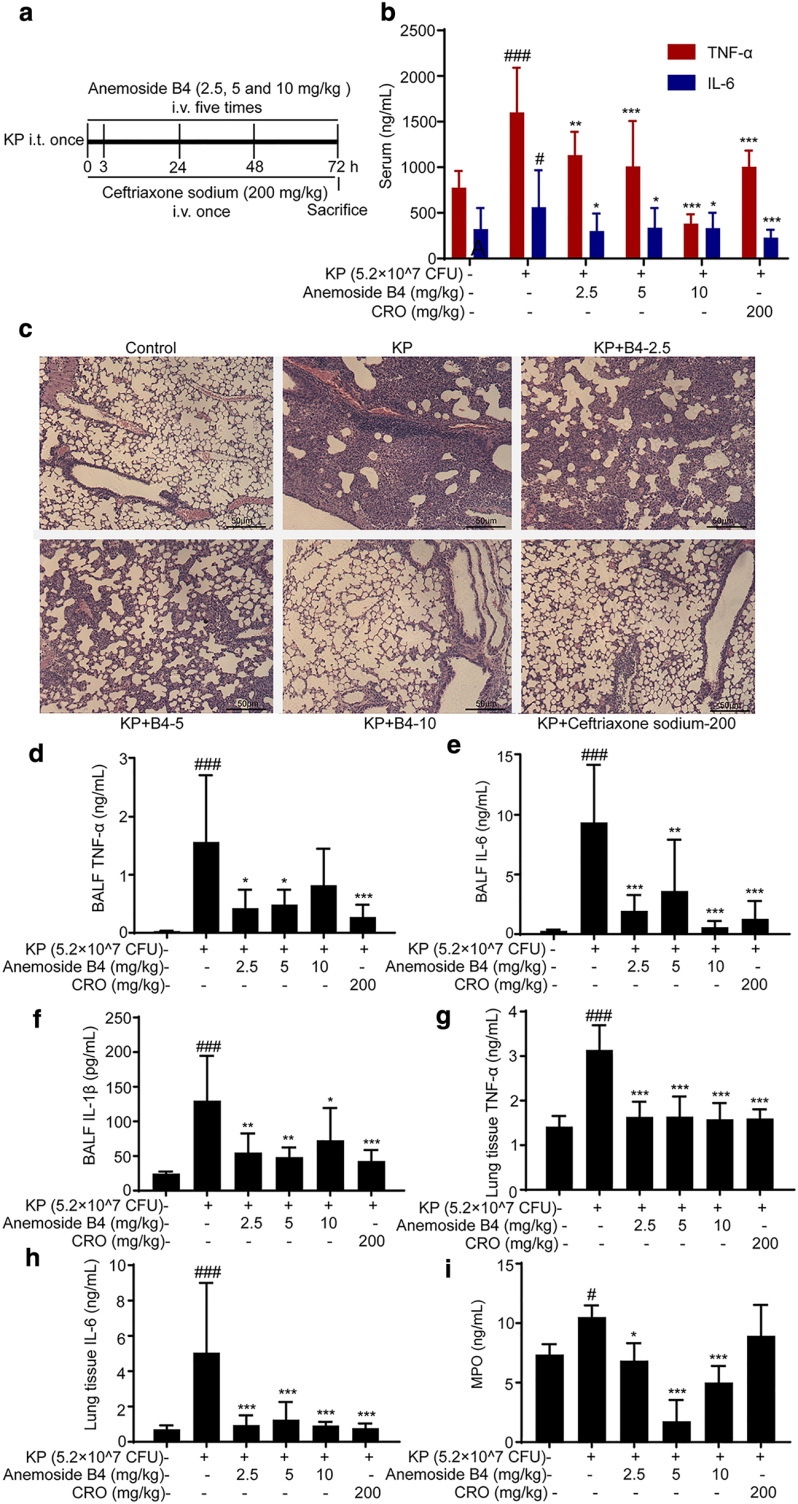
Effects of B4 on pro-inflammatory cytokines. **a** Experimental design of the bacterial pneumonia mouse model. The levels of TNF-α and IL-6 (**b**, **d**, **e**, **g**, **h**), IL-1β (**f**), and MPO (**i**) levels in serum, BALF and lung tissue were determined by ELISA kits and MPO kit. **c** The lung tissue inflammatory changes in each group were assessed by H&E staining of the lung slices and examined. Scale bar 50 μm. Value represents mean ± SD (n = 10), ^#^p < 0.05, ^###^p < 0.001 vs. control group, *p < 0.05, **p < 0.01, ***p < 0.001 vs. KP group

Next, we investigated the effect of anemoside B4 on pro-inflammatory cytokines in vivo. Guided by the prediction results, we explored the effects of anemoside B4 on the three cytokines. The results indicated that infection of KP caused a significant increase in the secretions of TNF-α and IL-6 (Fig. 3b) in mouse serum. Then, the administration of B4 (from 2.5 to 10 mg/kg) reduced the production of these cytokines. HE staining of lung tissue results showed that compared with the control group, KP induced obvious consolidation, alveolar damage, collapse, obvious compression, blurred border and obvious inflammatory cell infiltration; the lung tissue damage in the B4 treatment group significantly improved, alveolar collapse was significantly improved, consolidation was significantly relieved, the boundary was clearer, and compression and inflammatory changes were significantly reduced, of which the highest dose was the most obvious. In addition, significant increase was observed in the TNF-α (Fig. 3d, g), IL-6 (Fig. 3e, h), IL-1β (Fig. 3f), and MPO (Fig. 3i) in the BALF and lung tissue samples collected from the KP-infected pneumonia mice, which were reversed by anemoside B4. Collectively, anemoside B4 significantly prevents KP-induced pneumonia.

### Anemoside B4 restores KP-induced imbalance of blood parameters

**Fig. 4:**
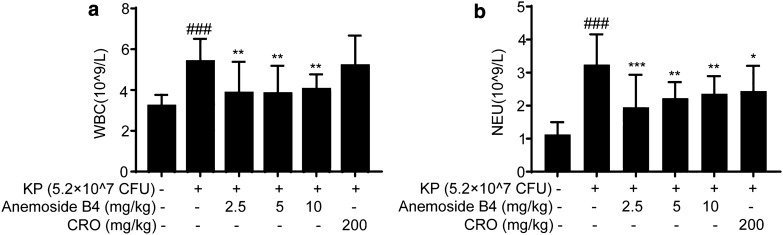
Effects of anemoside B4 on blood parameters. **a** The counts of white blood cells (WBC). **b** The counts of neutrophils (NEU). Value represents mean ± SD (n = 20), ^###^p < 0.001 vs. control group, *p < 0.05, **p < 0.01, ***p < 0.001 vs. KP group

The blood counts of WBC and NEU are the commonly-use biomarkers of bacterial pneumonia clinically [16]. Direct contact of the lungs with *Klebsiella pneumoniae* can easily cause lung inflammation in mice. The blood counts of WBC (Fig. 4a) and NEU (Fig. 4b) in the KP group were dramatically increased compared with control group. However, anemoside B4 suppressed the aberrant elevation of WBC and NEU respectively, of which the effects similar to the positive drug ceftriaxone sodium.

### Anemoside B4 decreases FM1-induced cytokines release in serum

**Fig. 5:**
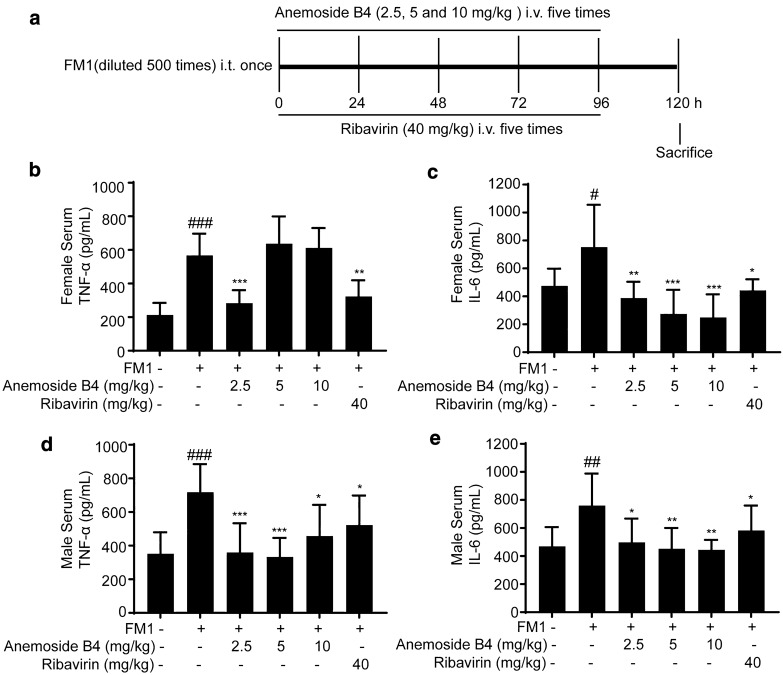
Effects of B4 on serum parameters of mice infected by FM1. **a** Experimental design of the viral pneumonia mouse model. **b** The level of TNF-α in female mice serum. **c** The level of IL-6 in female mice serum. **d** The level of TNF-α in male mice serum. **e** The level of IL-6 in male mice serum. Value represents mean ± SD (n = 10), ^#^p < 0.05, ^##^p < 0.01, ^###^p < 0.001 vs. control group, *p < 0.05, **p < 0.01, ***p < 0.001 vs. FM1 group

Influenza virus FM1-infected pneumonia leads to an inflammatory storm, suggesting that many pro-inflammatory cytokines like TNF-α and IL-6 released into blood and lung tissue [28–30]. In our study, FM1 was non-invasive intratracheally instilled into lung tissue of mice. Results showed that mice infected by FM1 caused a significant rise in the secretions of TNF-α (Fig. 5b, d) and IL-6 (Fig. 5c, e) in both female and male mouse serum, which was suppressed by B4. Thus, anemoside B4 exhibited a protective effect on FM1-induced pneumonia.

### Anemoside B4 decreased inflammatory response of lung tissue of mice infected by FM1

**Fig. 6:**
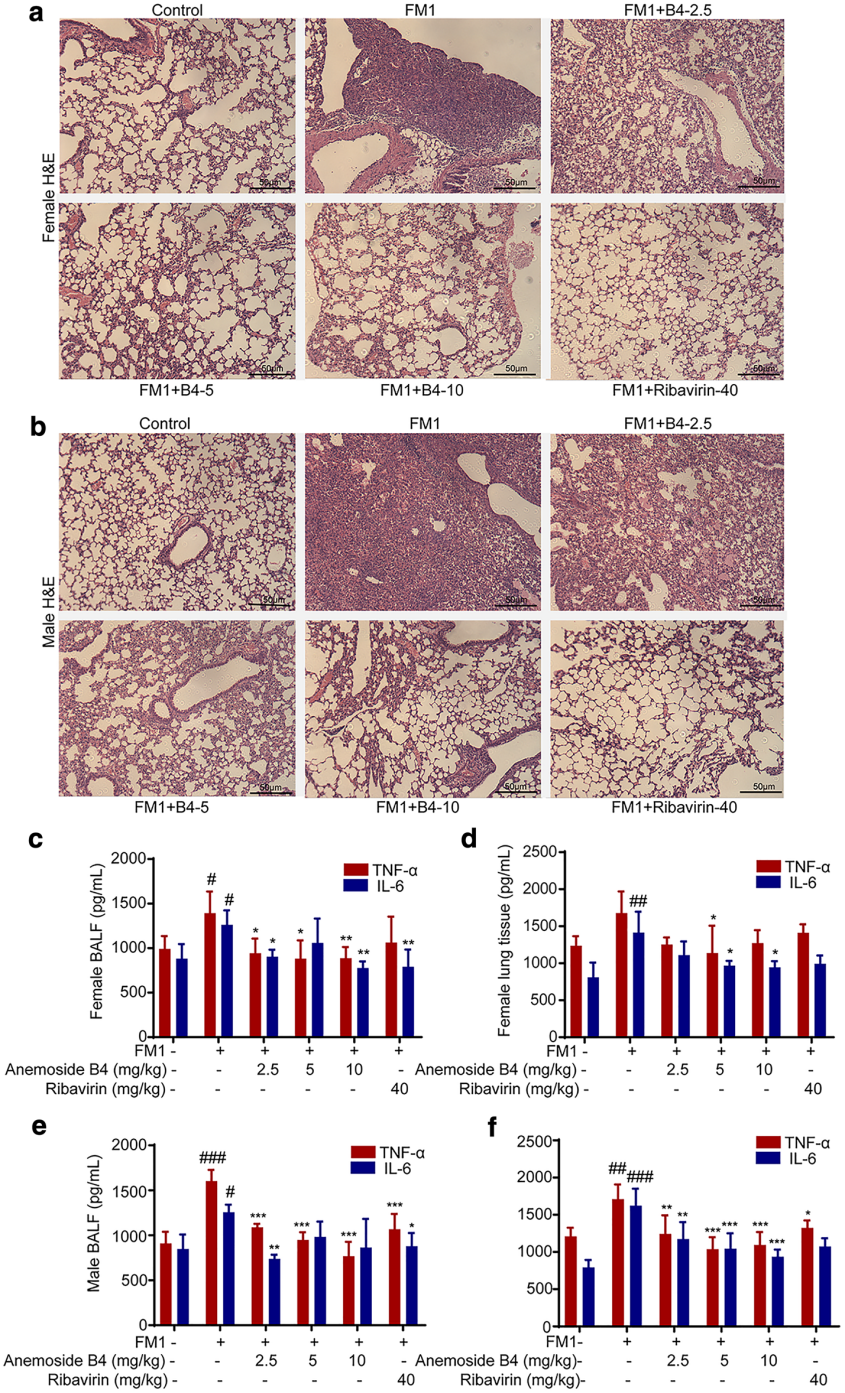
Effects of B4 on lung parameters with FM1 infection. HE staining detected pathological changes in lung tissue of female and male mice (**a**, **b**). The level of TNF-α, IL-6 (**c**) in female mice BALF, the level of TNF-α, IL-6 (**d**) in female mice lung tissue, the level of TNF-α, IL-6 (**e**) in male mice BALF, The level of TNF-α, IL-6 (**f**) in male mice lung tissue were determined by ELISA kits. Scale bar 50 μm. Value represents mean ± SD (n = 5), ^#^p < 0.05, ^##^p < 0.01, ^###^p < 0.001 vs. control group, *p < 0.05, **p < 0.01, ***p < 0.001 vs. FM1 group

To assess the protective effect of anemoside B4 on lung inflammation induced by FM1 infection, the levels of TNF-α and IL-6 were examined in BALF and lung tissue and HE staining of lung tissue was observed. As shown in Fig. 6, there were no obvious pathological changes in lung tissues of mice in the control group; pathological changes of lung tissues in the FM1 group showed edema, structural disorder, thickened alveolar septum, alveolar cavity shrinkage, and inflammatory cell infiltration. After treatment with B4 or ribavirin, the pathological changes of lung tissue were significantly alleviated in both female and male mice (a, b). The levels of TNF-α and IL-6 (Fig. 6c–f) in BALF and lung tissue were significantly increased by FM1 infection, which was reversed by anemoside B4. Our study indicated that anemoside B4 ameliorated FM1-induced lung inflammation, of which the effects were similar to the positive drug ribavirin.

### Anemoside B4 prevents FM1-induced pneumonia via TLR4/MyD88 pathway

**Fig. 7:**
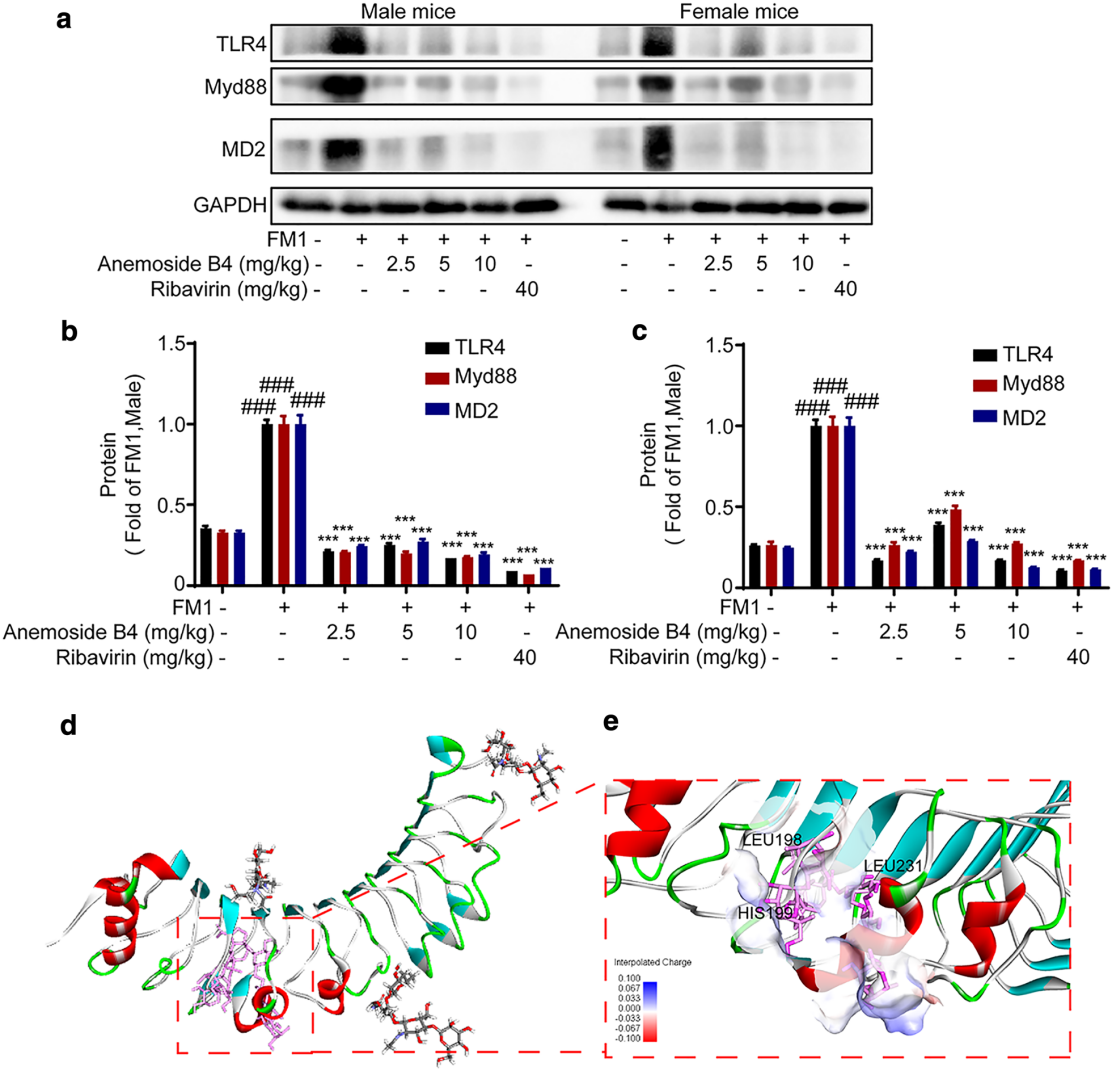
Effects of B4 on the TLR4/MyD88 pathway. **a**–**c** The protein expression of TLR4, MyD88, and MD2 were measured by western blotting. The mixed mice proteins were used in this experiment. **d**, **e** Docking results of B4 with TLR4. The chemical structure of B4 is shown in pink. TLR4 is shown in other colors. Value represents mean ± SD (n = 5), ^###^p < 0.001 vs. control group, ***p < 0.001 vs. FM1 group

TLR4, a transmembrane receptor located on the surface of many cells, plays a pivotal role in inflammatory processes [31]. Once stimulated, TLR4 forms a dimer and then regulates the downstream protein, thereby spawning a pathogen-specific innate immune response through releasing pro-inflammatory cytokines [32, 33]. To further study the anti-inflammatory mechanism of anemoside B4, the expression of TLR4, Myd88, and MD2 were analyzed in lung tissues. The results showed that FM1 activated the expression of TLR4, MyD88, and MD2, which was significantly suppressed by B4 (Fig. 7a–c). Furthermore, molecular docking assays showed that B4 was bound to TLR4 (Fig. 7d) and interacted with several amino acid sites including LEU198, LEU231 and HIS199 (Fig. 7e), which occupied the space and weakened the activation of TLR4 by FM1. Taken together, anemoside B4 ameliorated FM1-induced pneumonia via the TLR4/MyD88 pathway with binding to TLR4.

## Discussion

Pneumonia is inflammation of the terminal airways, alveoli, and interstitial lungs, which can be caused by pathogenic microorganisms such as bacteria, viruses, fungi, and atypical pathogens [34]. Among them, bacteria and virus are the main pathogenic factors [35]. Bacterial factors including streptococcus pneumoniae, staphylococcus aureus, and *Klebsiella pneumoniae*, etc. leads to pneumonia [36]. Although antibiotics were found to effectively decrease the death induced by bacterial-induced pneumonia, yet the abuse of antibiotics leads to drug-resistant or super bacterial, which is a worldwide problem. In addition to bacterial, virus like coronavirus, influenza virus, cytomegalovirus, etc. always lead to pneumonia [37]. Specifically, COVID-19 virus-induced pneumonia has a higher mortality. So far, there are not effective drugs for the treatment of COVID-19-pneumonia. Thus, to explore an effective drug to treat with pneumonia induced by bacterial or virus is imperative and necessary.

*Pulsatilla chinensis* (Bunge) Regel, a traditional Chinese medicine, was commonly used in the treatment of a variety of infectious diseases and malignant tumors [23]. A variety of saponins with anti-inflammatory and anti-tumor effects have been isolated from the root of *Pulsatilla chinensis* (Bunge) Regel, among which anemoside B4 is the major ingredient that quantized over 4.6% in terms of 2015 edition Chinese Pharmacopoeia. As it stands now, quite few studies on the pharmacological activity of anemoside B4 were found. For example, Hu et al. found that anemoside B4 could alleviate intestinal dysfunction by reducing inflammatory reaction [38, 39], which has therapeutic effects on immune system-related diseases [40, 41]. Anemoside B4 can reduce the nephrotoxicity of cisplatin [42] and the renal damage caused by adenine [43]. However, there is no relevant report on the effect and mechanism of anemoside B4 on pulmonary inflammation. In this study, we investigated the effects of B4 on pneumonia.

Data analysis from network pharmacology shows that B4 has a potential for regulating pro-inflammatory cytokines such as IL-1β, IL-6, and TNF-α. Among which, TNF-α is an important pro-inflammatory cytokines in the body, which plays a vital role in various physiological responses such as the synthesis and release of inflammatory mediators, neutrophil accumulation in the lungs, and complement activation [44]. And IL-6, a key cytokine produced by activated T cells and fibroblasts, has a wide range of biological activities such as immunoregulation [45, 46]. Studies have shown that IL-6 can catalyze and amplify the inflammatory response and its expression level effectively reflects the severity of tissue cell damage, which is used as an effective indicator for clinical diagnosis of acute and chronic inflammation [47]. IL-1β is an important inflammatory factor with dual sources of peripheral and central nerve, which can induce production and release of various inflammatory factors such as IL-8 [48]. Myeloperoxidase (MPO), a hemoglobin protein that is rich in neutrophils, can catalyze the oxidation of chloride ions to produce hypochlorous acid, kill microorganisms in phagocytic cells, and destroy various target substances [49]. It plays a key role in the body to produce and regulate inflammatory response. Hematology analysis is an effective way to detect pneumonia, especially pneumonia caused by bacterial infection [50]. In most cases, a significant increase in white blood cells and neutrophils can be observed in bacterial pneumonia [51]. Therefore, hematology counts and pro-inflammatory cytokines were selected as test indicators. In practical experiments, anemoside B4 exerted significant inhibitory effects on MPO, IL-1β, IL-6, and TNF-α. Besides, anemoside B4 decreased WBC and Neu cell counts in blood of the pneumonia mice. Therefore, anemoside B4 has a significant protective effect on KP- or FM1-infected pneumonia in vivo.

Toll-like receptor 4 (TLR4), the first identified member of TLR family, is a transmembrane protein characterized by an extracellular domain containing leucine-rich repeats (LRRs) with which the MD-2 molecule is associated [52]. After activated by virus, the TIR domain of TLR4 interacts with the TIR domain of MyD88 and binds to another TIR-containing adaptor protein, MyD88 adaptor-like (MAL), leading to an inflammatory cascade effector enzyme such as the expression of TNF-α, IL-1β, and IL-6 [53–56]. Thus, when B4 bound to TLR4, it will prevent the virus from activating TLR4, which may be an alternative strategy to treat FM1-induced viral pneumonia. Previous study indicated that emodin can inhibit influenza viral-induced pneumonia via the TLR4 pathway [57]. In this study, our results indicated that B4 inhibited FM1-induced expression of TLR4/MyD88/MD2 proteins. Furthermore, our docking results indicated that B4 could directly bind to TLR4 protein. Therefore, anemoside B4 suppressed the FM1 or KP-induced pneumonia via the TLR4/Myd88 pathway.

## Conclusion

**Fig. 8:**
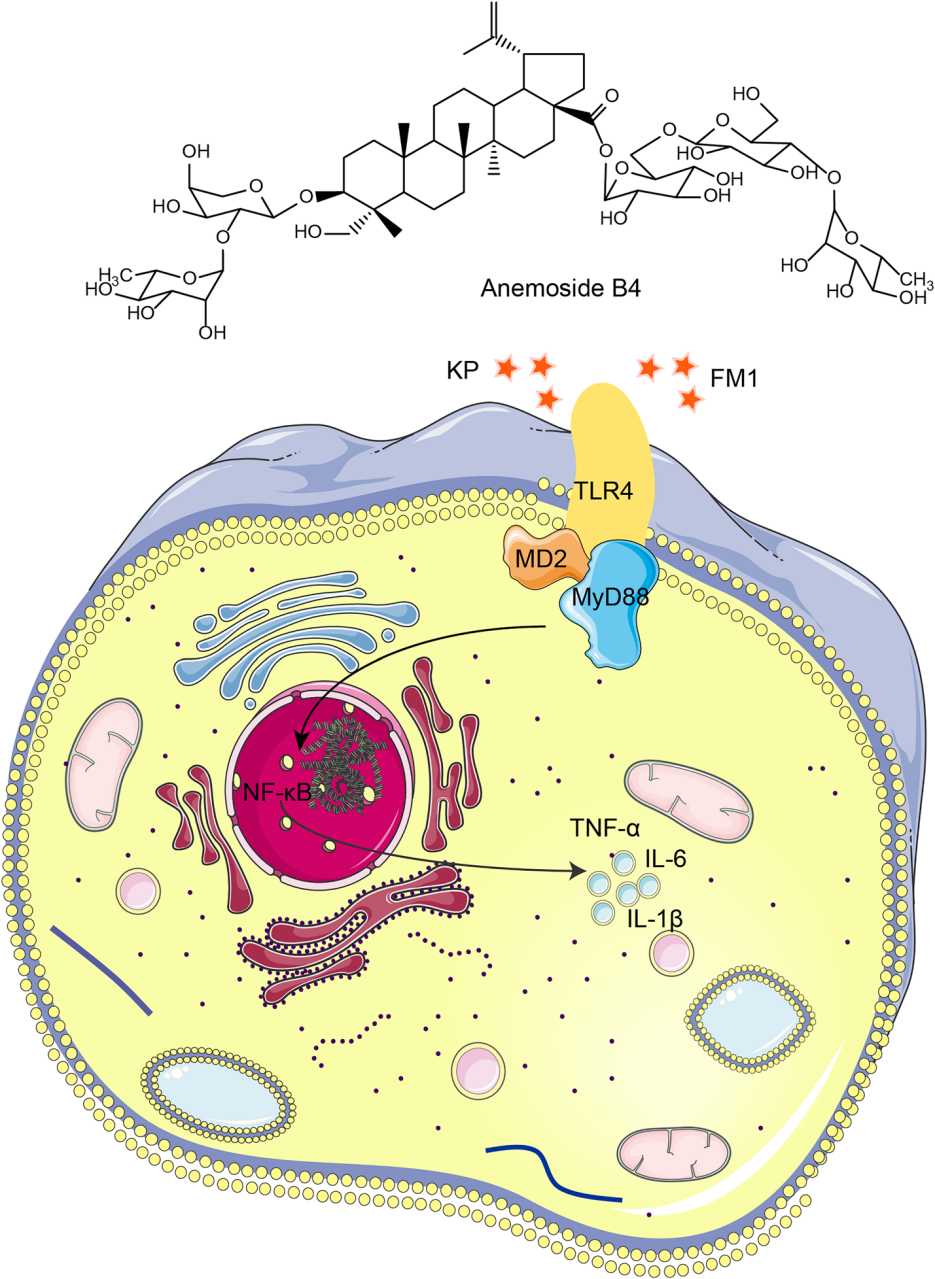
The schematic of anemoside B4’s anti-inflammatory mechanism

In summary, our study demonstrated that anemoside B4 exhibited significant protective effects on *Klebsiella pneumoniae*- or influenza virus FM1 induced pneumonia via theTLR4/Myd88 signaling pathway (Fig. 8).

## Data Availability

Not applicable.

## Abbreviations

B4: Anemoside B4
KP: *Klebsiella pneumoniae*
FM1: Influenza virus FM1
WBC: White blood cell
NEU: Neutrophils
MPO: Myeloperoxidase
IL-1β: Interleukin-1β
IL-6: Interleukin-6
TNF-α: Tumor necrosis factor-α
TLR4: Toll-like receptor 4
Myd88: Myeloid differentiation factor 88
MD2: Myeloid differentiation protein-2
BALF: Bronchoalveolar lavage fluid
